# A mitogenomic approach to the taxonomy of pollocks: *Theragra chalcogramma *and *T. finnmarchica *represent one single species

**DOI:** 10.1186/1471-2148-7-86

**Published:** 2007-06-07

**Authors:** Anita Ursvik, Ragna Breines, Jørgen Schou Christiansen, Svein-Erik Fevolden, Dag H Coucheron, Steinar D Johansen

**Affiliations:** 1 Department of Molecular Biotechnology, Institute of Medical Biology, University of Tromsø, N-9037 Tromsø, Norway; 2 Department of Aquatic BioSciences, Norwegian College of Fishery Science, University of Tromsø, N-9037 Tromsø, Norway; 3 Department of Fisheries and Natural Sciences, Bodø University College, N-8049 Bodø, Norway

## Abstract

**Background:**

The walleye pollock (*Theragra chalcogramma*) and Norwegian pollock (*T. finnmarchica*) are confined to the North Pacific and North Atlantic Oceans, respectively, and considered as distinct species within the family Gadidae. We have determined the complete mtDNA nucleotide sequence of two specimens of Norwegian pollock and compared the sequences to that of 10 specimens of walleye pollock representing stocks from the Sea of Japan and the Bering Sea, 2 specimens of Atlantic cod (*Gadus morhua*), and 2 specimens of haddock (*Melanogrammus aeglefinus*).

**Results:**

A total number of 204 variable positions were identified among the 12 pollock specimens, but no specific substitution pattern could be identified between the walleye and Norwegian pollocks. Phylogenetic analysis using 16.500 homologous mtDNA nucleotide positions clearly identify the Norwegian pollock within the walleye pollock species cluster. Furthermore, the Norwegian pollock sequences were most similar to mitochondrial genotypes present in walleye pollock specimens from the Sea of Japan, an observation supported both by neighbor-joining, maximum parsimony, and maximum likelihood analyses.

**Conclusion:**

We infer that walleye pollock and Norwegian pollock represent one single species and that Norwegian pollock has been recently introduced from the Pacific to the Atlantic Oceans.

## Background

The walleye pollock (*Theragra chalcogramma*) is a commercially important codfish species confined to the North Pacific Ocean from the Sea of Japan to the Gulf of Alaska [1]. The population structure of walleye pollock has been investigated by the use of various genetic markers including allozymes, microsatellites, and mitochondrial DNA sequences. The structuring is still unclear despite identification of distinct stocks in geographic regions including Sea of Japan, Sea of Okhotsk, Bering Sea, and Gulf of Alaska [2-5]. The Norwegian pollock (*T. finnmarchica*) is a very rare codfish species that was first discovered and described in 1932, and with a geographical distribution restricted to coastal regions of northern Norway for all the ca 50 specimens so far recorded [6]. The Norwegian pollock closely resemble the walleye pollock, but previous comparative examinations of morphological features have concluded that the two pollocks represent distinct species [7,8].

Mitogenomics is a high-resolution molecular genetic approach that includes the complete mitochondrial genome sequence (ca 16.500 bp) in the analyses. Mitogenomics, combined with molecular phylogenetics, has successfully resolved controversial issues of the origin and genetic variation of modern humans [9,10], as well as phylogenetic relationships among closely related fish species [11,12]. In the present study we have performed analysis that includes the complete mitochondrial genome sequences from multiple individuals of Norwegian pollock (*T. finnmarchica*), walleye pollock (*T. chalcogramma*), Atlantic cod (*Gadus morhua*) and haddock (*Melanogrammus aeglefinus*) in order to resolve the controversial relationship of the *Theragra *species.

## Results

**Figure 1:**
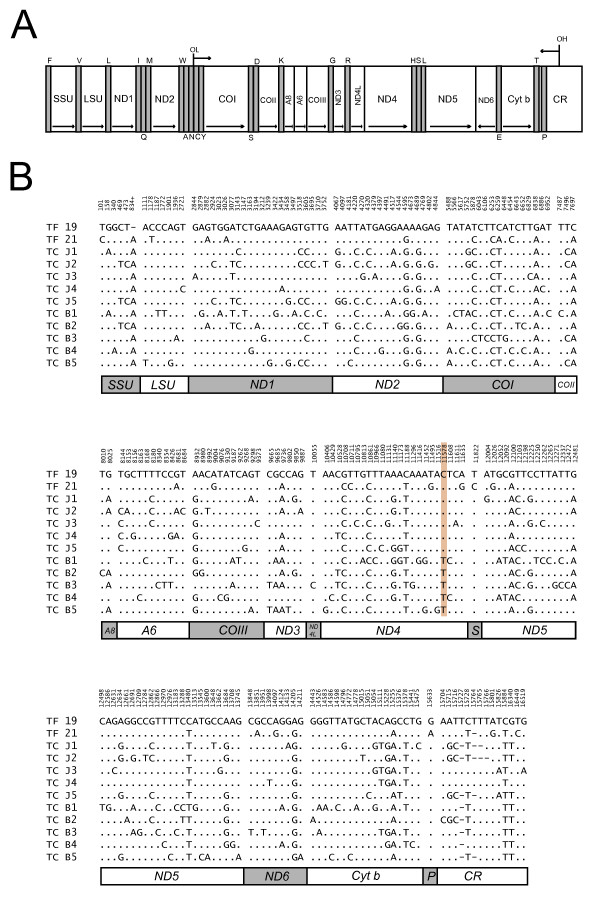
**Gene content, organization, and variability of *Theragra *mitochondrial genomes**. (**A**) Linear map of the circular mtDNA. All genes, except ND6 and 8 of the transfer RNA genes (indicated by the standard one-letter symbols for amino acid below the diagram), are encoded by the H-strand. Abbreviations: SSU and LSU, mitochondrial small- and large-subunit ribosomal RNA genes; ND1-6, NADH dehydrogenase subunit 1 to 6; COI-III, cytochrome c oxidase subunit I to III; A6 and A8, ATPase subunit 6 and 8; Cyt b, cytochrome b; OH and OL, origin of H-strand and L-strand replication; CR, control region containing the D-loop. (**B**) Distribution of variable sites in pollock mtDNA numbered according to the sequence of the Norwegian pollock Tf 19 (AM489718, Table 1). The position 834+ in SSU rDNA indicates nucleotide insertion between positions 834 and 835 in Tf 19. The variable sites were aligned to that of Tf 19. Identical sites are indicated by dots and deletions by dashes. The proposed diagnostic single nucleotide polymorphism at ND4 of Sea of Japan and Bering Sea pollocks [14] is boxed at position 11578.

The complete mitochondrial genome sequence was determined for two individuals of Norwegian pollock (*T. finnmarchica*), as well as one new individual each of Atlantic cod (*G. morhua*) and haddock (*M. aeglefinus*) (Table 1). The mtDNAs were approximately 16.6 kb in length with identical gene content and organization (13 protein coding genes, 2 ribosomal RNA genes, and 22 transfer RNA genes; Fig 1A) compared to previously published sequences in Atlantic cod, walleye pollock and haddock [13-15]. Heteroplasmy was detected in the ETAS (extended termination associated sequence) region within the control region in both Atlantic cod and Norwegian pollock. Atlantic cod mtDNA contains a heteroplasmic tandem repeat (HTR) motif of 40 bp that vary in copy number from 2–5 [16,17]. Heteroplasmy at single sites corresponding to that observed in walleye pollock [14] was also found in Norwegian pollock mtDNA. Thus, the ETAS region was excluded in subsequent phylogenetic analyses.

**Table 1:** Key features of Gadidae specimens and complete mtDNA sequences

**Name**	**Specimen; Location**	**mtDNA accession no.**	**Reference**
*Theragra finnmarchica *(Norwegian pollock)	Tf 19; Norwegian coastal	AM489718	This work
*T. finnmarchica*	Tf 21; Norwegian coastal	AM489719	This work
*T. chalcogramma *(Walleye pollock)	J1; Sea of Japan	AB182300	[14]
*T. chalcogramma*	J2; Sea of Japan	AB182301	[14]
*T. chalcogramma*	J3; Sea of Japan	AB182302	[14]
*T. chalcogramma*	J4; Sea of Japan	AB182303	[14]
*T. chalcogramma*	J5; Sea of Japan	AB182304	[14]
*T. chalcogramma*	B1; Bering Sea	AB094061	[14]
*T. chalcogramma*	B2; Bering Sea	AB182305	[14]
*T. chalcogramma*	B3; Bering Sea	AB182306	[14]
*T. chalcogramma*	B4; Bering Sea	AB182307	[14]
*T. chalcogramma*	B5; Bering Sea	AB182308	[14]
*Gadus morhua *(Atlantic cod)	NC1; Norwegian coastal	X99772	[13]
*G. morhua*	NF1; Newfoundland	AM489716	This work
*Melanogrammus aeglefinus *(Haddock)	NS; North Sea	DQ020497	[15]
*M. aeglefinus*	No1; Norwegian coastal	AM489717	This work

Nucleotide substitutions and deletions were assessed by comparing the complete mtDNA sequence of the 12 pollock specimens. The total number of variable sites identified was 204, and include all protein coding and ribosomal RNA genes, the control region (D-loop), and 2 of the 22 transfer RNA genes (Fig 1B). Transition substitutions at third codon positions of protein coding genes were the most common changes, and nucleotide deletions were only observed at one site in the SSU rRNA gene as well as in the ETAS region of the control region. However, no specific substitution feature could be identified in any of the Norwegian pollock sequences in comparison to that of the walleye pollock sequences, including no unique sites that distinguished the two proposed pollock species. Yanagimoto et al. [14] identified a single nucleotide polymorphism in the ND4 gene as a diagnostic marker of pollocks from the Sea of Japan and the Bering Sea. Interestingly, both the Norwegian pollock specimens harbour the Sea of Japan-type of nucleotide at this position (C11578 in the TF 19 sequence, Fig 1B).

A total of 16.500 nucleotide positions were unambiguously aligned from 16 specimens representing Norwegian pollock (2 specimens), walleye pollock (10 specimens), Atlantic cod (2 specimens), and haddock (2 specimens). The two specimens of Atlantic cod and haddock were selected in order to represent distant geographic locations (eastern and western Atlantic for *G. morhua*, and North Sea and northern Norway for *M. aeglefinus*). Pair wise distance within and between species are given in Table 2. In all cases, except for the two pollock species, interspecific p-distances were found to be significantly higher (approx. 10×) than distances within a defined species. Here, p-distances among the Atlantic cod specimens and the haddock specimens are 0.005 and 0.009, respectively, which are similar to that of the 12 pollock specimens (range 0.002–0.005). The p-distances between genera are about 10 fold higher. This observation in the mitochondrial DNA sequence is consistent with the conclusion that Norwegian pollock and walleye pollock are not genetically distinct.

**Table 2:** Summary of p-distances between and within species based on 16.500 nt positions

**Species**	**No. specimens**	**P-distance (range)**
*Theragra finnmarchica*	2	0.002
*T. chalcogramma*	10	0.002–0.005
*Gadus morhua*	2	0.005
*Melanogrammus aeglefinus*	2	0.009
*T. finnmarchica*/*T. chalcogramma*	2/10	0.003–0.004
*Theragra*/*G. morhua*	12/2	0.039–0.041
*Theragra*/*M. aeglefinus*	12/2	0.082–0.083
*G. morhua*/*M. aeglefinus*	2/2	0.084

**Figure 2:**
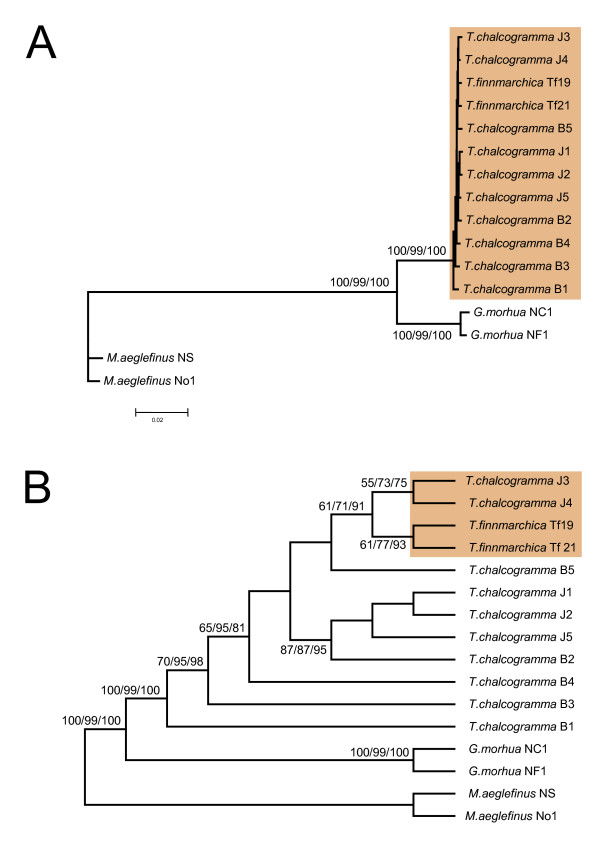
**Phylogenetic relationship of *Theragra***. (**A**) Maximum-likelihood (ML) phylogenetic tree based on 16.500 nucleotide positions and the TMV+I+G evolutionary model. Trees constructed by neighbour-joining (NJ, Jukes-Cantor substitution model) and maximum parsimony (MP, heuristic searches) displayed almost identical topologies with the ML tree. Bootstrap values (2000 replications) are shown at the branches (ML/MP/NJ). *Theragra *sequences are boxed. (**B**) Tree presenting the topology (same ML tree as in A) with bootstrap values (2000 replications) over 50% at the branches (ML/MP/NJ). The relationship between *T. finnmarchica *and the two speciemens J3 and J4 of *T. chalcogramma *from the Sea of Japan are boxed.

Phylogenetic analysis of the complete mitochondrial genome data-set using maximum likelihood (ML), Neighbor-joining (NJ), and maximum parsimony (MP), all resulted in a very similar tree topology (Fig. 2) with high statistical supports. Interesting findings noted from the complete mtDNA analysis are that the two Norwegian pollock sequences cluster together with those of walleye Pollock, and that the two Norwegian pollock sequences are more closely related to walleye pollock sequences isolated from specimens in the Sea of Japan (J3 and J4) than any of the specimens from the Bering Sea. From the mitogenomic phylogeny analysis we infer that Norwegian pollock and walleye pollock have to be considered as one single species. The data implicates that the genus *Theragra *consists of only one species confined to both the North Atlantic and North Pacific Oceans, a situation similar to that proposed for the Pacific cod (*G. macrocephalus*) and Greenland cod (*G. ogac*) based on partial mtDNA sequence analysis [18]. Although the genetic data are unequivocal, recent and extensive morphological examination of *Theragra *suggest that the formal taxonomic designations are upheld, but at the subspecific level (SEF, unpublished results).

## Discussion

Our finding that the Norwegian pollock appears recently derived from walleye pollock lineages within the Sea of Japan, is puzzling. One possibility is that a subpopulation of walleye pollock migrated from the North Pacific Ocean into the North Atlantic Ocean through the Bering Strait and Arctic Ocean, the only plausible connection between the Pacific and Atlantic Oceans in the Northern Hemisphere. If so, the Norwegian pollock would be expected to be more genetically similar to walleye pollock from the Bering Sea than that from the Sea of Japan due to geographical distances. However, this assumption is not supported in our analysis. Furthermore, walleye pollock has not been reported in the Arctic Ocean, between the Bering Strait and coast of Norway, despite intense fishery activities over years [19]. Alternatively, walleye pollock could have been introduced (intentionally or unintentionally) by human to the northeast Atlantic from the Sea of Japan. In fact, the Soviet Russian authorities did some experiments around 1930 on transfer of marine species by railway from Vladivostok to Murmansk (see ), but currently there are no available documentations that include walleye pollock. Whatever the reason may be, the presence of a small population of pollock in the North Atlantic Ocean is a very interesting observation and should be included in ecosystem monitoring approaches [20] of the Arctic Ocean.

## Conclusion

Based on the complete mitochondrial genome sequences we conclude that the walleye pollock (*Theragra chalcogramma*) and Norwegian pollock (*T. finnmarchica*) represent one single species, and that Norwegian pollock has been recently introduced from the Pacific to the Atlantic Oceans.

## Methods

### Fish samples and DNA extraction

Specimens of *T. finnmarchica *(Tf. 19 and Tf. 21) were collected at May 11, 2003 and April 29, 2003, respectively, in Norwegian coastal waters east of the North Cape. The *G. morhua *specimen was collected off Newfoundland, Canada (NF1), and the *M. aeglefinus *specimen (No1) was collected off the coast of northern Norway. Key-features of fish samples and mitochondrial DNA sequences used in this study are listed in Table 1. DNA was extracted from muscle tissue by using the mtDNA Extractor CT Kit from Wako. The method makes use of differential centrifugation steps to obtain a crude isolation of the mitochondria in the membrane fraction, lysis of the mitochondria, and subsequent precipitation of the supernatant with sodium iodide and isopropanol.

### PCR amplification, cloning, and DNA sequencing

Primers (Table 3) designed from our published Atlantic cod mtDNA sequence (X99772) [13] were used to amplify 1 – 4 kb fragments using one heavy (H) and one light (L) strand primer. Each PCR reaction (25 μl) included solution, 0.2 mM dNTPs, 0.2 μM of each primer and 0.6 U of Expand HiFidelity polymerase and buffer (Roche) in addition to the total DNA sample. The PCR reactions were performed in a Peltier 200 Thermal cycler with the following cycling parameters: 94°C initial denaturation for 2 min, 30 cycles with 94°C denaturation for 15 sec, 53°C annealing for 30 sec, 68°C elongation for 2 – 3 min, and finally 72°C for 7 min. The total volumes were run on 0.8 or 1% agarose gels containing ethidium bromide, and bands were excised and purified with Qiagen gel extraction kit. When appropriate, PCR products were inserted into the pDrive vector (Qiagen) and transformed in *E. coli *EZ competent cells. Positive clones were verified with plasmid isolation (Wizard plasmid purification kit) and *Eco*RI restriction cutting followed by agarose gel electrophoresis. In general, PCR products were sequenced on both strands by using the BigDye version 3.1 kit (Applied Biosystems) with the same primers as in the PCR and internal primers (Table 3). The reaction mixture included 10 – 60 ng of the PCR-template, 0,35 μM primer, 2 μl BigDye mix, 1 μl 5× reaction buffer in a 10 μl volume. The sequencing products were analysed by an ABI genetic analyser (Applied Biosytems).

**Table 3:** PCR and DNA sequencing primers

**Primer**	**Sequence (5', 3')**
L42	GAT GGA CCC TAG AAA GTC C
H171	AGA TGT GCC TGA TAC CTG CT
L290	GAA AGC TTG ACT TAG TTA AG
H414	TGA CTT CGG ATG CGT ATA AC
H617	TAG AAC AGG CTC CTC TAG
L1223	CGC AAG GGA ACG CTG AAA
H1275	AGG TAC GAG TAG AAA ACT CTG
L1757	CTT ACC AGG CTG TCT TAT GC
H1805	GTC CGT TCC GAC TTA CAC
L2222	ATT ACA TAA GAC GAG AAG AC
L3089	GCC AGT ACT TGC ACT AAC TC
H3562	AGC CCA GAA ATA GTA CAG CT
L3760	TGG CAC TAG TGA TTT GAC AT
L4366	GTA CAC TTC TGG TTA CCA GA
H4559	AGC CAA GAT GTG CGA TTG AT
L4876	TAA GCC TTT ACT TTT ATC T
L5232	CTC TTA GTT AAC AGC TAA GC
H5475	AGG GTG CCA ATG TCT TTG TG
L5572	TCG AGC AGA GCT AAG TCA AC
L6604	ATG TAT AGG AGC TGT CTT TG
H6880	TCA ACT GCT ATT ACT TCC CG
L7208	ATC ACC CGT AAT AGA AGA GT
L7901	TGG AAG CAG GTG ACT CCC AA
H8439	ATG ACC TAG TGC ATG AGT TG
L8575	TTA CAG CTA ATC TTA CAG CA
L9302	TTC AAG GAC TGG AGT ACT AT
H10545	TGA CTT GCA AGG AGT ATT AG
L11407	GAC CAC ATG ATG ATT TAT TG
L10603	TAT AAA CCG CCA ACG TA
H11714	ATC TAA TGT CTT GGT TA
H11887	ACT TGG AGT TGC ACC AAG AG
L12121	TAT AGA GGC TGT AAC TTC TT
H13913	GTG AGT ACC TGT AGA TGA GT
L14331	CCA CCG TTG TTA TTC AAC T
H14746	AAT TAC GGT AGC TCC TCA GAA TGA TAT TTG TCC TCA
L15246	TAT TCT CCA TTC TAG TCC TT
L15250	CTC GAT TCT AGT CCT CAT GG
L15500	ACT GAG CTA CTA GGG CAG TTT C
H15666	GTT TAA TTT AGA ATT CTA GCT TTG G
H16180	GAA TAG CCA GGA AAC GTG TTA
H16390	AAC CGA GGA CTA GCT CCA CC
SP6	GATTTAGGTGACACTATAG
T7	AATACGACTCACTATAG

### Data analysis

In general, computer analyses of DNA sequences were performed using software package programs from DNASTAR Inc. For the phylogenetic analyses, nucleotide sequences based on 16.500 positions covering the complete mitochondrial genomes except the highly variable ETAS, were used to make a multiple alignment as one single dataset using ClustalX version 1.81 [21] and manual refinements. MEGA version 3.1 [22] was used to estimate pairwise distances using the uncorrected p-distance model. Furthermore, MEGA was used to construct trees with the methods of neighbor joining (NJ) using different distance matrices, and maximum parsimony (MP) with heuristic searches using close-neighbor-interchang (CNI) level 3 and production of initial trees by of random addition of sequences (100 replicates). Maximum likelihood (ML) analyses, based on the sequence evolution model TMV+G+I selected by the program WinModeltest version 4b [23], were conducted with PAUP* (version 4.0 b10) [24]. The reliability of tree branching points was assessed by bootstrapping (2000 replications).

## Authors' contributions

AU and RB did the sequencing of the mitochondrial genomes. DHC performed the phylogenetic analysis. DHC, SDJ, AU, and RB contributed to mtDNA sequence analyses. JSC and SEF contributed with fish samples and valuable discussions. SDJ directed the research and wrote the paper in collaboration with DHC.

## References

[B1] Bailey KM, Stabeno PJ, Powers DA (1997). The role of larval retention and transport features in mortality and potential gene flow of walleye pollock. J Fish Biol.

[B2] Bailey KM, Quinn TJ, Bentzen P, Grant WS (1999). Population structure and dynamics of walleye pollock, *Theragra chalcogramma*. Adv Mar Biol.

[B3] Olsen JB, Merkouris SE, Seeb JE (2002). An examination of spatial and temporal genetic variation in walleye pollock (*Theragra chalcogramma*) using allozyme, mitochondrial DNA, and microsatellite data. Fishery Bulletin.

[B4] O'Reilly PT, Canino MF, Bailey KM, Bentzen P (2004). Inverse relationship between Fst and microsatellite polymorphism in the marine fish, walley pollock (*Theragra chalcogramma*): implications for resolving weak population structure. Mol Ecol.

[B5] Grant WS, Spies IB, Canino MF (2006). Biogeographic evidence for selection on mitochondrial DNA in North Pacific walleye pollock *Theragra chalcogramma*. J Heredity.

[B6] Christiansen JS, Fevolden S-E, Byrkjedal I (2005). The occurrence of *Theragra finnmarchica *Koefoed 1956 (Teleostei, Gadidae), 1932–2004. J Fish Biol.

[B7] Koefoed E (1956). *Theragra finnmarchica*, n. sp. A fish caught off Berlevåg allied to the Alaskan pollock, *Theragra chalcogramma *Pallas from the Bering Sea. Fiskeridirektoratets Skrifter Serie Havundersøkelser.

[B8] Svetovidov AN (1959). On the occurrence of the genus *Theragra *in the Barents Sea in connection with some problems of the derivation of the amphiboreal gadoids and clupeoids. Zoological Journal.

[B9] Ingman M, Kaessmann H, Paabo S, Gyllensten U (2000). Mitochondrial genome variation and the origin of modern humans. Nature.

[B10] Tanaka M, Cabrera VM, Gonzalez AM, Larruga JM, Takeyasu T, Fuku N, Guo LJ, Hirose R, Fujita Y, Kurata M, Shinoda K, Umetsu K, Yamada Y, Oshida Y, Sato Y, Hattori N, Mizuno Y, Arai Y, Hirose N, Ohta S, Ogawa O, Tanaka Y, Kawamori R, Shamoto-Nagai M, Maruyama W, Shimokata H, Suzuki R, Shimodaira H (2004). Mitochondrial genome variation in eastern Asia and the peopling of Japan. Genome Res.

[B11] Doiron S, Bernatchez L, Blier PU (2002). A comparative mitogenomic analysis of the potential adaptive value of Arctic charr mtDNA introgression in brook charr populations (*Salvelinus fontinalis *Mitchill). Mol Biol Evol.

[B12] Minegishi Y, Aoyama J, Inoue JG, Miya M, Nishida M, Tsukamoto K (2005). Molecular phylogeny and evolution of the freshwater eels genus *Anguilla *based on the whole mitochondrial genome sequences. Mol Phylogenet Evol.

[B13] Johansen S, Bakke I (1996). The complete mitochondrial DNA sequence of Atlantic cod (*Gadus morhua*): relevance to taxonomic studies among codfishes. Mol Mar Biol Biotechnol.

[B14] Yanagimoto T, Kitamura T, Kobayashi T (2004). Complete nucleotide sequence and variation of mitochondrial DNA from 10 individuals of walleye Pollock, *Theragra chalcogramma*. Fisheries Science.

[B15] Roques S, Fox CJ, Villasana MI, Rico C (2006). The complete mitochondrial genome of the whiting, *Merlangius merlangius*, and the haddock, *Melanogrammus aeglefinus*: a detailed genomic comparison among closely related species of the Gadidae family. Gene.

[B16] Johansen S, Guddal PH, Johansen T (1990). Organization of the mitochondrial genome of Atlantic cod, *Gadus morhus*. Nucleic Acids Res.

[B17] Arnason E, Rand DM (1992). Heteroplasmy of short tandem repeats in mitochondrial DNA of Atlantic cod, *Gadus morhua*. Genetics.

[B18] Coulson MW, Marshall HD, Pepin P, Carr SM (2006). Mitochondrial genomics of gadine fishes: implications for taxonomy and biogeographic origins from whole-genome data sets. Genome.

[B19] Andriyashev AP, Chernova NV (1995). Annotated list of fishlike vertebrates and fish of the Arctic Sea and adjacent waters. Journal of Ichthyology.

[B20] Smetacek V, Nicol S (2005). Polar ocean ecosystems in a changing world. Nature.

[B21] Thompson JD, Gibson TJ, Plewniak F, Jeanmougin F, Higgins DG (1997). The ClustalX windows interface: flexible strategies for multiple sequence alignment aided by quality analysis tools. Nucleic Acids Res.

[B22] Kumar S, Tamura K, Nei M (2004). MEGA3: Integrated software for Molecular Evolutionary Genetics Analysis and sequence alignment. Briefings in Bioinformatics.

[B23] Posada D, Crandall KA (1998). Modeltest: testing the model of DNA substitution. Bioinformatics.

[B24] Swofford DL (2002). PAUP*. Phylogenetic analysis using parsimony (and other methods). Version 4.

